# Perceived Breast Cancer Risk among Female Undergraduate Students in Ghana: A Cross-Sectional Study

**DOI:** 10.1155/2021/8811353

**Published:** 2021-04-16

**Authors:** Eric Osei, Sandra Osei Afriyie, Samuel Oppong, Emmanuel Ampofo, Hubert Amu

**Affiliations:** ^1^Department of Population and Behavioural Sciences, School of Public Health, University of Health and Allied Sciences, PMB 31, Hohoe, Ghana; ^2^Department of Public Health Graduate School, Yonsei University, Seoul, Republic of Korea; ^3^Department of Physician Assistantship, School of Medicine, University of Health and Allied Sciences, PMB 31, Hohoe, Ghana; ^4^Department of Epidemiology and Biostatistics, School of Public Health, University of Health and Allied Sciences, PMB 31, Hohoe, Ghana; ^5^Human Resource Division, University of Cape Coast, Cape Coast, Ghana

## Abstract

**Background:**

While breast cancer accounts for the highest mortality among women across the globe, little is known about its perceived risks among them. We examined the perceived risk of breast cancer among undergraduate female university students in Ghana.

**Methods:**

This was a cross-sectional survey of 358 undergraduate female students at the University of Health and Allied Sciences, Ghana. Descriptive and inferential statistics comprising frequencies, percentages, chi-square, and binary logistic regression analyses were used in analysing the data collected. All analyses were done using STATA Version 13.1.

**Results:**

Seventy-three percent were aware of breast cancer and 45.2% out of this did not perceive themselves to be at risk of breast cancer. Academic year (*p*=0.02), school (*p*=0.01), knowledge of someone with breast cancer (*p* < 0.001), family history of breast cancer (*p* < 0.001), current use of oral pills/injectable contraception (*p*=0.03), history of breast cancer screening (*p* < 0.001), and intention to perform breast self-examination (*p* < 0.001) were the risk factors of breast cancer risk perception. Students without a family history of breast cancer were 90% less likely to perceive breast cancer risk (AOR = 0.10, 95% CI = 0.04–0.29) compared with those having a family history of breast cancer. Students who had never screened for breast cancer were also 62% less likely to perceive that they were at risk of breast cancer (AOR = 0.10, 95% CI = 0.04–0.29) compared with those who had ever screened for breast cancer.

**Conclusion:**

This study showed that female university students tend to estimate their breast cancer risk based on their experience of breast cancer. Students who have ever screened for breast cancer and those with the intention to perform breast self-examination in the future are more likely to perceive themselves as being at risk and thus take action to avoid getting breast cancer.

## 1. Introduction

Globally, breast cancer has become the leading cancer among women with about 2.1 million cases each year and 627,000 deaths in 2018 [1]. In Ghana, the disease is increasingly becoming a considerable public health problem. According to the international agency for cancer research, breast cancer is the most common cancer in Ghana with approximately 4,700 new cases diagnosed and more than 1,800 deaths occurring in 2018 [1]. The incidence of breast cancer is expected to increase as Ghana's population ages and women adopt western lifestyles [2, 3].

Research has shown that breast cancer screening remains a useful tool for reducing breast cancer mortality among women [4, 5]. However, misunderstanding of breast cancer and its related risk factors, as well as the erroneous perception of one's own risk, may have an adverse influence on screening behaviours [6]. Risk perception is an essential subjective psychological phenomenon related to threat evaluation that is closely linked to one's judgment about vulnerability to disease and the likelihood of benefit from taking a preventive action [7]. It is considered as a key construct in several theories of health behaviour, such as the health belief model, the precaution adoption model, the transtheoretical model of stress coping, and the protection motivation theory [8–10].

Risk perception is recognized as a strong driving force for women to engage in preventive and screening behaviours, yet previous studies suggest that most women incorrectly perceive their breast cancer risk [11, 12]. Women who do not perceive themselves as being vulnerable to breast cancer may not care much about the use of early diagnostic methods such as mammography [13, 14] and other important tools such as Breast Self-Examination (BSE) and Clinical Breast Examination (CBE). Notwithstanding that the effectiveness of BSE and CBE remains debatable [15], their possible contribution to early detection of breast abnormalities cannot be overlooked. BSE for instance may be a useful tool in resource-limited countries to detect any abnormality in the breast as it provides an opportunity for women to be familiar with their breast and promptly report any changes. Additionally, clinical encounters by women provide the opportunity for women to have a number of important clinical activities such as breast cancer risk assessment, education about lifestyle, counselling, and clinical breast examination that may not otherwise be done [16].

Since risk perception may be an important motivator for women to adopt preventive behaviours including breast screening, it is important to understand the predictors of perceived risk of breast cancer, which can be considered when planning interventions to alter health behaviours. The perception of risk, health beliefs, and attitudes towards breast cancer can affect the choice and use of early screening methods [15, 17, 18]. However, little is known about the factors associated with breast cancer risk perception in Ghana. This study, therefore, aimed to explore the perceived risk of breast cancer among female undergraduate students in Ghana and to examine factors associated with perceived risk. This population is especially important because they have abundant access to health information as health students and they are potential sources of information for non-health students and the general population. Additionally, college students have a significant influence on fellow students, and also, findings regarding this target group have implications for their capacity in the role of promoting screening for breast cancer as potential health professionals.

## 2. Methods

### 2.1. Study Site and Design

We conducted a cross-sectional survey among female undergraduate students at the University of Health and Allied Sciences (UHAS) of Ghana in April 2019. UHAS is the only public university dedicated to the training of healthcare professionals in Ghana. There are six academic schools and one institute in the university. The current study was conducted among students from the school of Nursing and Midwifery, the School of Pharmacy, the School of Medicine, the School of Allied Health Sciences, and the School of Basic and Biomedical Sciences. The university's current student population is about 3,750. UHAS offers eighteen undergraduate programmes including Medicine, Physician Assistantship, Nursing, Midwifery, Physiotherapy, Pharmacy, Dietetics, Speech and Language Therapy, Disease Control, etc. [19].

### 2.2. Sampling

A sample size of 385 female students was estimated using a single proportion formula: *n* = *z*^2^(*pq*)/*d*^2^ where *n* is the needed sample size; *z*, the degree of accuracy at 95% confidence level; *p* = the estimated proportion of an attribute that is present in the population; *q* = 1 − *p*; and *d* = margin of error. We assumed a margin of error (*d*) of 0.05 at 95% confidence level and an estimated proportion of 50%, since to the best of literature search made, there was no study done in the study area. Stratified sampling was used to select the study participants from the various academic years of study and different schools to ensure representativeness. The number of students selected from each academic year and school was proportional to the students' population.

### 2.3. Data Collection Tools and Procedures

A structured pretested questionnaire was used to collect data from participants. The questionnaire contained closed-ended questions. “Perceived risk of breast cancer” was the main outcome variable. Participants were asked to respond “yes,” “no,” or “do not know” to the following question: “Do you think that you have a chance of developing breast cancer?” Sociodemographic variables comprising age, marital status, religion, ethnicity, the academic year of study, and school were examined as explanatory variables. Breast cancer risk factors including age, family history of breast cancer, age at menarche, and age at first live birth also constituted explanatory variables in the study. The others were alcohol consumption, hormonal contraception use, and physical activity. Screening-related factors consisted of a history of breast cancer screening and the intention to perform BSE in future. The questionnaires were self-administered after explaining the study's objectives to them. The filled questionnaires were checked by the researchers together with the participants for consistency and omission before collection. Where there were inconsistencies, participants were made to correct them in the presence of the researchers before they were taken. Participants were given 24 hours to return filled questionnaires to have enough time to fill them.

### 2.4. Data Analysis

Descriptive statistics were used to describe the study population in relation to relevant variables. Chi-square analysis was used to test for statistical significance between sociodemographic and risk factors of breast cancer and perceived risk, which was the main outcome variable. Variables significant at *p* value <0.05 in the bivariate analysis were included in a multivariate binary regression analysis. The degree of association between dependent and independent variables was assessed using adjusted odds ratios (AOR) with 95% confidence intervals (CIs). All analyses were done using STATA statistical package Version 13.1 from Stata Corp.

## 3. Results

### 3.1. Sociodemographic Characteristics of Respondents

Table 1 presents the sociodemographic characteristics of respondents. Fifty-five percent were 20 and 24 years old. Students constituting 83.1% were never married, 74% were Christians, and 38.4% were Akans. First-year students were 26.2%, second-year students constituted 21.8%, 30.2% were in the third year, and 21.8% were fourth-year students. The majority (52.5%) belonged to the School of Nursing and Midwifery, 22.3% were in the School of Medicine, and 19.7% were studying Allied Health Sciences (Table 1).

### 3.2. Breast Cancer Risk Factors

Table 2 presents breast cancer risk factors among the respondents. Menarche occurred at below 12 years old in 12.5% of participants. The prevalence of current use of oral pills/injectable was 13.2%. A family history of breast cancer was confirmed by 14.3% of the respondents; 4% had their first-degree relatives (mother, sister) affected, and 11% had their second-degree relatives affected with breast cancer. Regarding behavioural factors, 36% did not engage in regular physical activity, and 20% took at least one standard drink of alcohol per day (Table 2).

### 3.3. Breast Cancer Awareness and Risk Perception

Table 3 presents breast cancer awareness and risk perception among the respondents. Seventy-three percent of the students had ever heard of breast cancer. Out of this, 45.2% did not consider themselves at risk of breast cancer. “I take good care of myself” (45.7%), I do regular breast self-examination” (*n* = 23.6%), and I have no family history of breast cancer (30.7%) were the main reasons for perceiving as not being at risk of breast cancer (Table 3).

### 3.4. Predictors of Breast Cancer Risk Perception

Table 4 presents the sociodemographic factors associated with breast cancer risk perception. Breast cancer risk perception insignificantly increased with age (*p*=0.67) but significantly increased with an increasing year of study (*p*=0.02). Additionally, the proportion of students who perceived themselves to be at risk of breast cancer was higher (71.4%) among students in the school of Basic and Biomedical Sciences, followed by the School of Medicine (55.2%) and School of Nursing and Midwifery (36.9%), but lowest among Pharmacy (16.7%) and Allied Health Sciences students (23.6%) (*p*=0.01). Academic year (*p*=0.02), school (*p*=0.01), knowledge of someone with breast cancer (*p* < 0.001), family history of breast cancer (*p* < 0.001), current use of oral pills/injectables (*p*=0.03), history of breast cancer screening (*p*<0.001), and intention to perform breast self-examination (*p* < 0.001) were the risk factors of breast cancer risk perception (Table 4).

Table 5 presents a multivariate logistic regression of predictors of breast cancer risk perception. Students without a family history of breast cancer were 90% less likely to perceive breast cancer risk (AOR = 0.10, 95% CI = 0.04–0.29, *p* < 0.001) compared with those having a family history of breast cancer. Women who had never screened for breast cancer were also 62% less likely to perceive that they were at risk of breast cancer (AOR = 0.10, 95% CI = 0.04–0.29, *p* < 0.001) compared with those who had ever screened for breast cancer. Students without any intention to perform breast self-examination in the future were as well 83% less likely to perceive breast cancer risk (AOR = 0.17, 95% CI = 0.04–0.29, *p* < 0.001) than those with an intention.

## 4. Discussion

For an individual to adopt any healthy behaviour, he/she should be able to feel vulnerable to a particular threat and must perceive the threat as serious, thus taking actions to salvage him/herself from that condition [20, 21]. This study was carried out to examine the extent to which Ghanaian female undergraduate university students perceived their risk of breast cancer. It also examined which characteristics were related to the students' perception of their own risk of breast cancer. In this regard, common risk factors of breast cancer, as well as breast screening variables (ever screened for breast cancer and the intention of future breast self-examination), were incorporated.

Predictably, the study demonstrates a significant level of optimism in breast cancer risk perception among Ghanaian female Undergraduates. Most of our study participants did not think they are vulnerable to breast cancer, and the most important reason underlying their optimism was “I take good care of myself.” The high rate of optimism towards breast cancer among this population could be due to the fact that they are relatively young as almost all of them (98.7%) were less than 30 years and might be aware of the fact that female breast cancer is most frequently diagnosed among women in advance age and as with most malignancies, the risk increases with age [22]. Notwithstanding, studies have shown that breast cancer is increasingly becoming common among younger Ghanaian women. Ohene-Yeboah and colleague for instance in their study at the Komfo Anokye Teaching Hospital in Kumasi, Ghana, found that nearly one-fourth of breast cancer cases occurred among women less than 40 years with a significant proportion of 4.5% occurring in women below 29 years [23]. Ghartey Jnr. and colleagues assessed the pattern and incidence of breast cancer in five regions of Ghana and demonstrated that more than 3 in 10 of all breast cancer cases identified in their screening programme were women below 30 years and over 4% were less than 25 years [24]. This is a clear demonstration that young Ghanaian women are increasingly becoming at risk of breast cancer and ought to perceive themselves as such. Notwithstanding ongoing health education and information about breast cancer in Ghana, women's understanding and interpretation of risk do not seem to be pertinent when it comes to their risk perception. Several previous studies have reported low-risk perception of breast cancer among women in different populations. Among South Korean women, nearly 70% of study participants perceived their breast cancer risk to be lower than others in the same age category [25]. In the Ogun state of Nigeria, only about 18% of rural and 15% of urban women believed that they could get breast cancer in their lifetime [13]. Fehniger et al. [26] reported in their multiethnic study in San Francisco that only 18% of high-risk women displayed optimism about their risk of breast cancer. In Turkey, Yavan et al. [14] demonstrated that about 50% of women believed that they were not likely to get breast cancer. Personal health risk perception is considered an important determinant of specific health-related behaviour. Regrettably, however, women, particularly of African origin, continue to ignore their chances of developing breast cancer in their lifetime. This optimism has negative implications on the health-seeking behaviours of women. Donnelly and colleagues [27], for instance, implicated low-risk perception among Arab women as a factor negatively influencing their disposition towards breast cancer screening, despite the rising incidence and mortality of the disease. Yavan et al. also reported that the increased cancer risk perception was associated with increased regular breast cancer screening or mammography use [14].

On the contrary, Constance et al. found a surprisingly inverse relationship between risk perception and willingness to screen for cervical cancer in women in Northern Ghana and postulated that fear of the unknown could deter women who think they are likely to be positive from accessing screening services [28]. Among Turkish women, Kartal et al. [6] also could establish any relationship between breast cancer risk perception and regular breast examination. Unexpectedly, however, the rate of optimism displayed by our study participants was far higher than what the previous study in Presbyterian University College in Ghana found, where 75% of female students in the School of Allied Health Sciences perceived themselves to be vulnerable to breast cancer [29]. The differences in the level of optimism displayed by women between the current study and the aforementioned study cannot directly be inferred. However, it could be related to differences in exposure to breast cancer information and education in the school and the community.

When we examined the relationship between breast cancer risk factors and perceived risk of breast cancer, family history of breast cancer, which can be perceived as a risk by directly experiencing the events, was the only risk factor associated with a higher perceived risk of breast cancer. Many studies in different locations and among different populations have shown that experience of breast cancer at a close range is associated with a higher odds of perceived risk of breast cancer [11, 25, 30]. These results confirm the findings that people with personal experience with health hazard are less likely to have optimistic biases [31, 32]. These results suggest that women who reported as having no risk of breast cancer might be basing that judgment, in part, on their family history of the disease, without considering other important risk factors that may influence the development of the disease, which may have a negative influence on their screening behaviour.

To establish whether the perceived risk of breast cancer predicts preventive behaviour, we examined the association between perceived risk and the intention to perform breast examination in future. Expectedly, the results showed that women who did not have an intention to perform breast cancer examination in future were 83% less likely to perceive themselves as being at risk of breast cancer, compared to those with the intention to perform breast examination in future. This confirms Park et al.'s [25] study which demonstrated that women with lower perceived comparative risk are more likely to have no intention of getting a mammogram.

## 5. Limitations

This study has two limitations. First, the findings cannot be generalized beyond the study population because they are young and well-educated women. University students are not representative of young adults in general, and the risk perception may differ from that of the general population. Second, all data were self-reported with no objective measures to assess the accuracy of these reports. However, the results of this study provide some understanding regarding the perceived risk and related factors of breast cancer among future health professionals, which can be useful for strategized communication and education.

## 6. Conclusion

This study shows a significant level of optimism in breast cancer risk perception among female undergraduate students in Ghana with an average risk of breast cancer. Only a family history of breast cancer that could be directly perceived through experience was found to be associated with a perceived risk of breast cancer. Furthermore, women who had previous breast cancer screening experience and those with the intention to perform a self-breast examination in the future positively perceived themselves as being at risk of breast cancer. We recommend periodic risk perception assessment among women of different birth cohorts in order to develop strategies that influence preventive behaviour among women.

## Figures and Tables

**Table 1:** Sociodemographic characteristics of respondents.

Variable	Frequency (*N* = 385)	Percent
Age (years)		
<20	105	27.3
20–24	215	55.8
25–29	60	15.6
30+	5	1.3

Marital status		
Married	65	16.9
Never married	320	83.1

Religion		
Christian	285	74.0
Muslim	73	18.9
Traditionalist	1	0.3
No religion	26	6.8

Ethnicity		
Akan	148	38.4
Ewe	119	30.9
Ga-Dangme	55	14.3
Others	63	16.4

Academic year		
First year	101	26.2
Second year	84	21.8
Third year	116	30.2
Fourth year	84	21.8

School		
Allied Health Sciences	74	19.7
Basic and Biomedical Sciences	7	1.8
Medicine	86	22.3
Nursing and Midwifery	202	52.5
Pharmacy	14	3.6

**Table 2:** Breast cancer risk factors among undergraduate female students.

Variable	*n*	%
Age at menarche (years)		
<12	48	12.5
≥12	337	87.5

Current use of oral pills/injectables		
Yes	51	13.2
No	334	96.8

Family history of breast cancer		
Yes	55	14.3
No	330	85.7

Number of first-degree relative with breast cancer		
0	371	96.0
≥1	14	4.0

Personal history of breast cancer		
Yes	4	1.0
No	381	99.0

Regular physical activity		
Yes	246	64.0
No	139	36.0

Current alcohol consumption (drink/day)		
0	308	80.0
≥1	77	20.0

**Table 3:** Breast cancer awareness and risk perception.

Variable	*N*	%
Have heard of breast cancer (*N* = 385)		
Yes	281	73.0
No	104	27.0

Breast cancer risk perception (*N* = 281)		
High risk	108	38.4
No risk	127	45.2
Unknown risk	46	16.4

Reason for not being at risk (*N* = 127)		
I take good care of myself	58	45.7
I do regular breast self-examination	30	23.6
I have no family history of breast cancer	39	30.7

**Table 4:** Perceived risk of breast cancer by sociodemographic characteristics.

Characteristics	Risk perception (*N* = 281)				
	Yes, *n*(%)	No, *n*(%)	Do not know, *N* (%)	*X* ^2^	*p* value
Age group (years)				4.07	0.67
<20	17 (38.6)	19 (43.2)	8 (18.2)		
20–24	64 (35.4)	87 (48.1)	30 (16.6)		
25–29	24 (47.1)	19 (37.3)	8 (15.7)		
≥30	3 (60.0)	2 (40.0)	0 (0.0)		

Religion				5.57	0.47
Christian	85 (38.1)	103 (46.2)	35 (15.7)		
Muslim	18 (40.9)	18 (40.9)	8 (18.2)		
Traditionalist	0 (0.0)	0 (0.0)	1 (100.0)		
No religion	5 (38.4)	6 (46.2)	2 (15.4)		

Ethnicity				6.32	0.39
Akan	38 (35.2)	55 (50.9)	15 (13.9)		
Ewe	36 (42.4)	38 (44.7)	11 (12.9)		
Ga-Dangbe	15 (34.1)	19 (43.2)	10 (22.7)		
Others	19 (43.2)	15 (34.1)	10 (22.7)		

Academic year				15.24	0.02
First year	17 (39.5)	12 (27.9)	14 (32.6)		
Second year	16 (32.6)	23 (46.9)	10 (20.4)		
Third year	39 (36.1)	55 (50.9)	14 (12.9)		
Fourth year	36 (44.4)	37 (45.7)	8 (9.8)		

School				16.68	0.01
Allied health sciences	13 (23.6)	27 (49.1)	15 (27.3)		
Basic and Biomedical Sciences	5 (71.4)	1 (14.3)	1 (14.3)		
Medicine	32 (55.2)	24 (41.4)	2 (3.45)		
Nursing and Midwifery	57 (36.8)	72 (46.5)	26 (16.8)		
Pharmacy	1 (16.7)	3 (50.0)	2 (33.3)		

Known someone with breast cancer				15.78	<0.001
Yes	70 (50.4)	54 (38.9)	15 (10.8)		
No	38 (26.8)	73 (51.4)	31 (21.8)		

Family history of breast cancer				35.89	<0.001
Yes	40 (74.1)	10 (18.5)	4 (7.4)		
No	68 (29.9)	117 (51.5)	42 (18.5)		

Alcohol consumption (drinks/day)				0.65	0.72
0	83 (37.4)	103 (46.4)	36 (16.2)		
≥1	25 (42.4)	24 (40.7)	10 (16.9)		

Current use of oral pills/injectables				7.07	0.03
Current user	16 (40.0)	23 (57.5)	1 (2.5)		
Non-user	92 (38.4)	104 (43.1)	45 (18.7)		

Physical activity				1.14	0.57
Yes	96 (37.5)	117 (45.7)	43 (16.8)		
No	12 (48.0)	10 (40.0)	3 (12.0)		

Age at menarche				1.22	0.54
<12	18 (46.2)	16 (41.0)	5 (12.8)		
≥12	90 (37.2)	111 (45.9)	41 (16.9)		

Ever screened for breast cancer				29.51	<0.001
Yes	81 (47.9)	75 (44.4)	13 (7.7)		
No	27 (24.1)	52 (46.4)	33 (29.5)		

Intention to perform BSE in future				43.46	<0.001
Yes	88 (78.6)	12 (10.7)	12 (10.7)		
No	19 (31.2)	33 (54.1)	9 (14.8)		

Known someone with breast cancer				15.78	<0.001
Yes	70 (50.4)	54 (38.9)	15 (10.8)		
No	38 (26.8)	73 (51.4)	31 (21.8)		

Family history of breast cancer				35.89	<0.001
Yes	40 (74.1)	10 (18.5)	4 (7.4)		
No	68 (29.9)	117 (51.5)	42 (18.5)		

Alcohol consumption (drinks/day)				0.65	0.72
0	83 (37.4)	103 (46.4)	36 (16.2)		
≥1	25 (42.4)	24 (40.7)	10 (16.9)		

Current use of oral pills/injectables				7.07	0.03
Current user	16 (40.0)	23 (57.5)	1 (2.5)		
Non-user	92 (38.4)	104 (43.1)	45 (18.7)		

Physical activity				1.14	0.57
Yes	96 (37.5)	117 (45.7)	43 (16.8)		
No	12 (48.0)	10 (40.0)	3 (12.0)		

Age at menarche				1.22	0.54
<12	18 (46.2)	16 (41.0)	5 (12.8)		
≥12	90 (37.2)	111 (45.9)	41 (16.9)		

Ever screened for breast cancer				29.51	<0.001
Yes	81 (47.9)	75 (44.4)	13 (7.7)		
No	27 (24.1)	52 (46.4)	33 (29.5)		

Intention to perform BSE in future				43.46	<0.001
Yes	88 (78.6)	12 (10.7)	12 (10.7)		
No	19 (31.2)	33 (54.1)	9 (14.8)		

**Table 5:** Multivariate logistic regression of predictors of breast cancer risk perception.

Variables	AOR [95% CI] *p* value
School	
Nursing and Midwifery	Ref.
Allied health sciences	1.33 [0.56–3.12] 0.513
Basic and Biomedical Sciences	1.70 [0.12–24.5] 0.696
Medicine	1.09 [0.50–2.37] 0.827
Pharmacy	6.54 [0.35–121.6] 0.208

Academic year	
First year	Ref.
Second year	0.91 [0.27–3.11] 0.884
Third year	0.48 [0.17–1.41] 0.186
Fourth year	0.52 [0.17–1.58] 0.249

Known someone with breast cancer	
Yes	Ref.
No	0.68 [0.35–1.36] 0.273

Family history of breast cancer	
Yes	Ref.
No	0.10 [0.04–0.29] <0.001

Current use of oral pill/injectables	
Current user	Ref.
Non-user	0.70 [0.28–1.74] 0.447

History of breast cancer screening	
Ever screened for breast examination	Ref.
Never screened for breast examination	0.38 [0.19–0.76] <0.01

Intention to perform breast self-examination in future	
Have the intention	Ref.
Have no intention	0.17 [0.09–0.35] <0.001

## Data Availability

The data used for this study can be obtained from the authors upon reasonable request.
